# Meta-analysis of the benefit of hypomethylating agents before allogeneic hematopoietic stem cell transplantation in myelodysplastic syndromes

**DOI:** 10.1007/s10238-021-00712-0

**Published:** 2021-04-17

**Authors:** Liu Liu, Menglu Jia, Ling Sun, Wenliang Tian, Ping Tang, Zhongxing Jiang

**Affiliations:** Department of Hematology, First Affiliated Hospital of Zhengzhou University, 1 Jianshe East Road, Zhengzhou, 450052 China

**Keywords:** Myelodysplastic syndromes, Hypomethylating agents, Allogeneic hematopoietic stem cell transplantation, Meta-analysis

## Abstract

Hypomethylating agents (HMAs) are effective therapies in myelodysplastic syndromes (MDS), but allogeneic hematopoietic stem cell transplantation (allo-HSCT) is the only way to cure MDS. According to the current literature, it is difficult to confirm whether HMAs bridging therapy is beneficial for MDS patients receiving allo-HSCT. Therefore, we tried to evaluate the effect of HMAs on long-term survival of the MDS patients. Databases, including PubMed, Embase Ovid, and the Cochrane Library, were searched for studies published up to January 10, 2021. Patients who accepted HMAs bridging to allo-HSCT were defined as experimental group, while patients who received the best supportive care (BSC) before allo-HSCT were control group. Overall survival (OS) was the primary end point. Seven studies were included in the final analysis. The final results showed no OS differences between patients accepted HMAs before allo-HSCT and those received BSC (HR = 0.86, 95% CI: 0.64–1.15, *p* = 0.32), indicating that MDS patients' long-term survival did not benefit from HMAs bridging therapy before allo-HSCT. This conclusion needs to be further verified by a large number of prospective randomized controlled trials, which have guiding significance for the treatment of MDS patients.

## Introduction

Myelodysplastic syndrome (MDS) is a group of heterogeneous clonal diseases originated from hematopoietic stem cells and characterized by myelodysplasia. Its clinical manifestations include anemia, infection and hemorrhage caused by hemocytopenia, and variable risk progressed to acute myeloid leukemia (AML).

Recent studies have shown that epigenetic changes, especially abnormal DNA methylation, are important factors leading to the occurrence and development of MDS [1–3]. HMAs, azacitidine (AZA) and decitabine (DEC), have been approved by FDA for MDS treatment since 2004. A number of studies have shown that HMAs are beneficial not only to improve the OS and leukemia transformation-free survival, but also reduce the infection rate and dependence of red blood cell transfusion of MDS patients [4–6]. Unfortunately, only 40%–50% of patients respond to HMAs treatment, and complete response rate is 10%–20%. Among clinical responders, the majority will experience loss of response and disease progression [7–9]. So, allo-HSCT is the only way to cure MDS to date, with a OS of 25%–52% [10–13]. However, HSCT candidates often need to wait several months, for the appropriate donor. Disease progression may result in losing the opportunity of transplantation, so the bridging treatment before allo-HSCT is particularly important. At present, common bridging therapies include demethylation therapy, conventional chemotherapy and the BSC.

The inhibition of DNA methyltransferase by HMAs is thought to be responsible for the hypomethylation and reactivation of tumor suppressor genes, inducing the terminal differentiation and apoptosis of neoplastic cells, which may contribute to improvements in allo-HSCT outcome by a reduction in tumor burden. And demethylation before allo-HSCT did improve the overall survival rate in some reports [14–18]. Alternatively, an increase in phenotypic expression during the differentiation and modification of leukemia cells may make them susceptible to immune surveillance mechanisms and result in their increased sensitivity to a GVL effect of allo-HSCT[19, 20]. Though exact mechanism is not clear, it seems rational for HMAs as a bridging therapy to allo-HSCT.

It is unclear, however, whether treatment with HMAs before allo-HSCT will increase the toxicity of the conditioning regimen or otherwise affect the results of transplantation, and whether patients with certain risk factors such as old age, complex karyotype, multi-gene mutations can benefit. Therefore, the purpose of this study is to explore the long-term efficacy of HMAs before allo-HSCT by integrating the clinical research in the current environment.

## Materials and methods

### Data sources and searches

The plan of this study was made in advance. We reported this meta-analysis based on the preferred reporting items for systematic reviews and meta-analyses. We did a full search using several databases: PubMed, EMBASE Ovid, and Cochrane Library (from database creation to January 10, 2021). We screened the abstracts and titles of articles eligible for further review. The full text of the research has been published and reviewed. The review of this meta-analysis has not been registered with Prospero.

### Inclusion and exclusion

The inclusion and exclusion criteria were as follows: (1) this study focused on the effect of HMAs on the prognosis of MDS patients before allo-HSCT; (2) in this study, bridging treatments before transplantation were HMAs and BSC; (3) sufficient clinical data were provided in this study, at least the OS; and (4) the hazard ratio (HR) and its 95% confidence interval (95% CI) were directly reported, or calculated from raw data; (5) the study was published in full in English; (6) the study included human subjects; (7) the article was not a review, case report or animal study. If the same or overlapping data were presented in multiple studies, only the most important or highest quality studies were included. Differences were resolved through discussion.

### Data extraction and outcome measures

Two reviewers independently extracted information from each eligible study and entered it into a spreadsheet. The data included the name of the first author, year of publication, country, time of inclusion, number of patients, age, HMAs classification, MDS subtype, MDS classification standard, bone marrow cell count, international prognostic scoring system (IPSS) score, human leukocyte antigen (HLA) matching, stem cell source and conditioning regime. We chose OS as the primary endpoint. OS was defined as the time from transplantation to the last follow-up of patients who died or survived. When HR doesn't have a report, we try to contact the author to get it, or use the method previously reported to calculate it.

### Methodologic quality and risk of bias

We used the Cochrane risk bias assessment tool to assess the bias risk in the trial, including the following areas: random sequence generation, allocation concealment, blinding of participants and personnel, blinding of outcome assessment, incomplete outcome data and selective reporting and other bias. Using the Cochrane risk bias assessment tool, bias risk can be assessed as low, unknown, or high. Two reviewers independently assessed the risk of bias in each study. Any conflict was resolved by consensus.

### Analysis

**Fig. 1:**
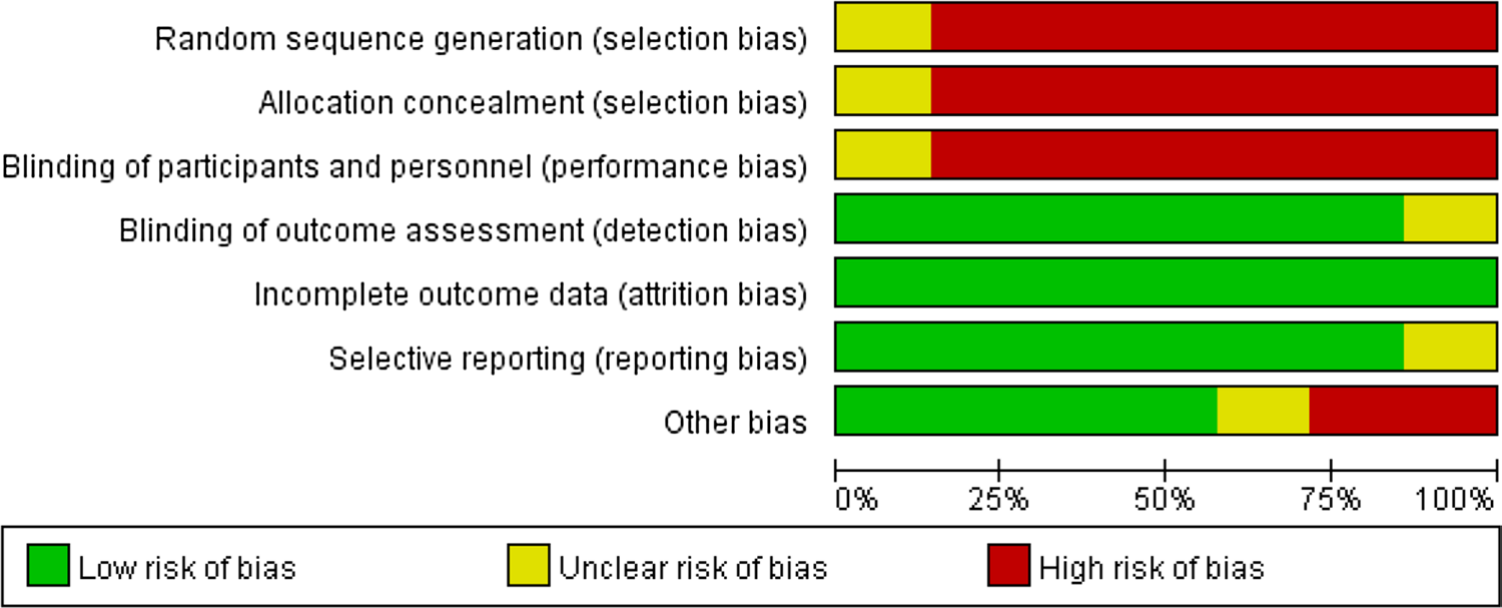
Risk of research bias

**Fig. 2:**
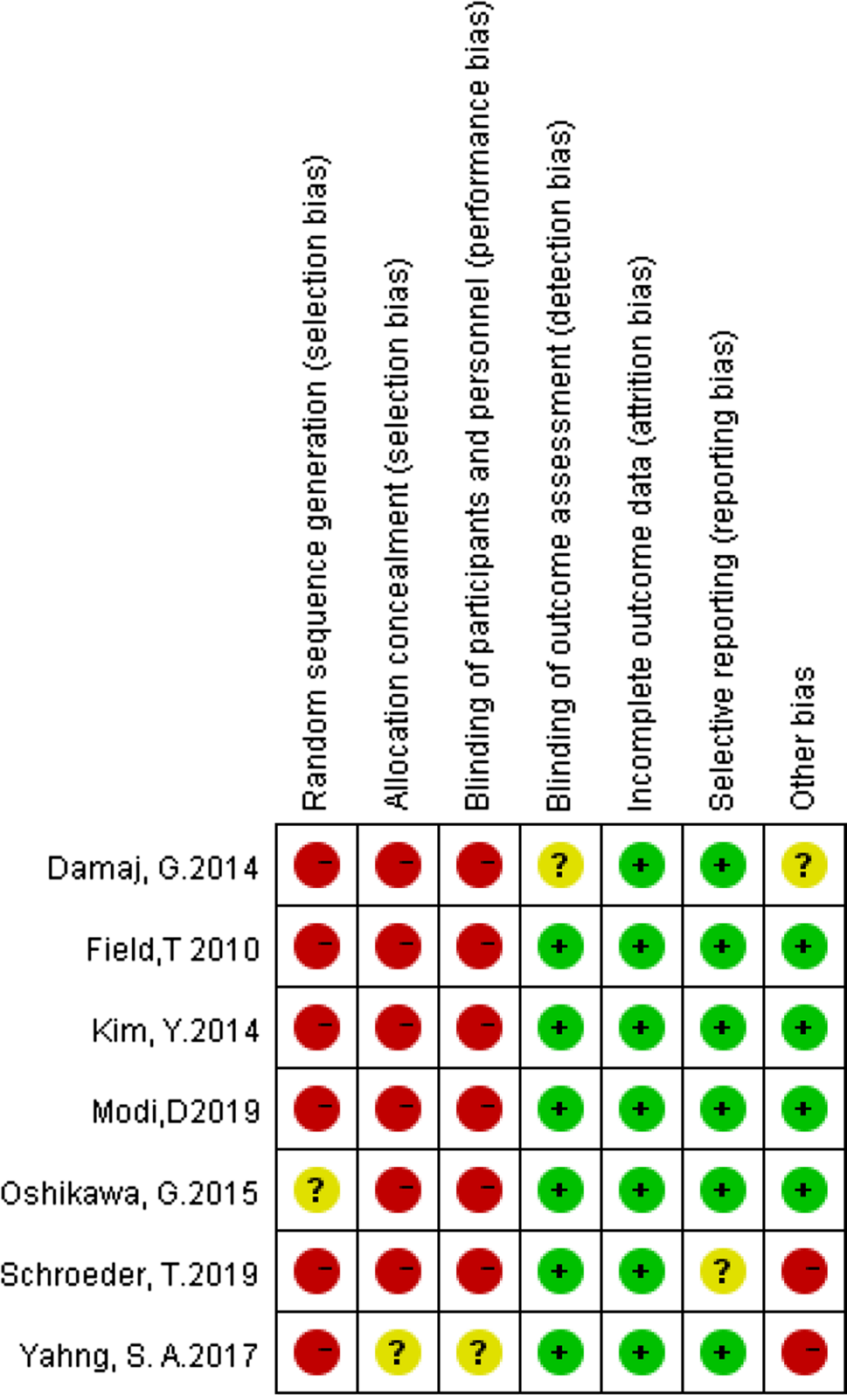
Summary of research bias risk

The calculation was carried out in stata12.0. By calculating HRs and its 95% CI, the influence of HMAs on OS of transplant patients was evaluated by general reverse variance method. *P* value less than 0.05 was considered statistically significant. Chi-square test was used to evaluate the heterogeneity of the study, and *P* value less than 0.10 was significant. We use the I^2 statistic to quantify heterogeneity. When the value of I^2 is less than 25%, between 25 and 50%, and more than 50%, the heterogeneity is considered as low, medium and high, respectively. If high heterogeneity is detected, a random effect model is used; otherwise, a fixed effect model is used for meta-analysis. We also use subgroup analysis to analyze the source of heterogeneity. The sensitivity analysis evaluates the stability of the combined results by the sequence omission of one study at a time. Funnel plot, Beck test and egger test were used to evaluate publication bias (Figs. 1 and 2

## Results

### Study selection

**Fig. 3:**
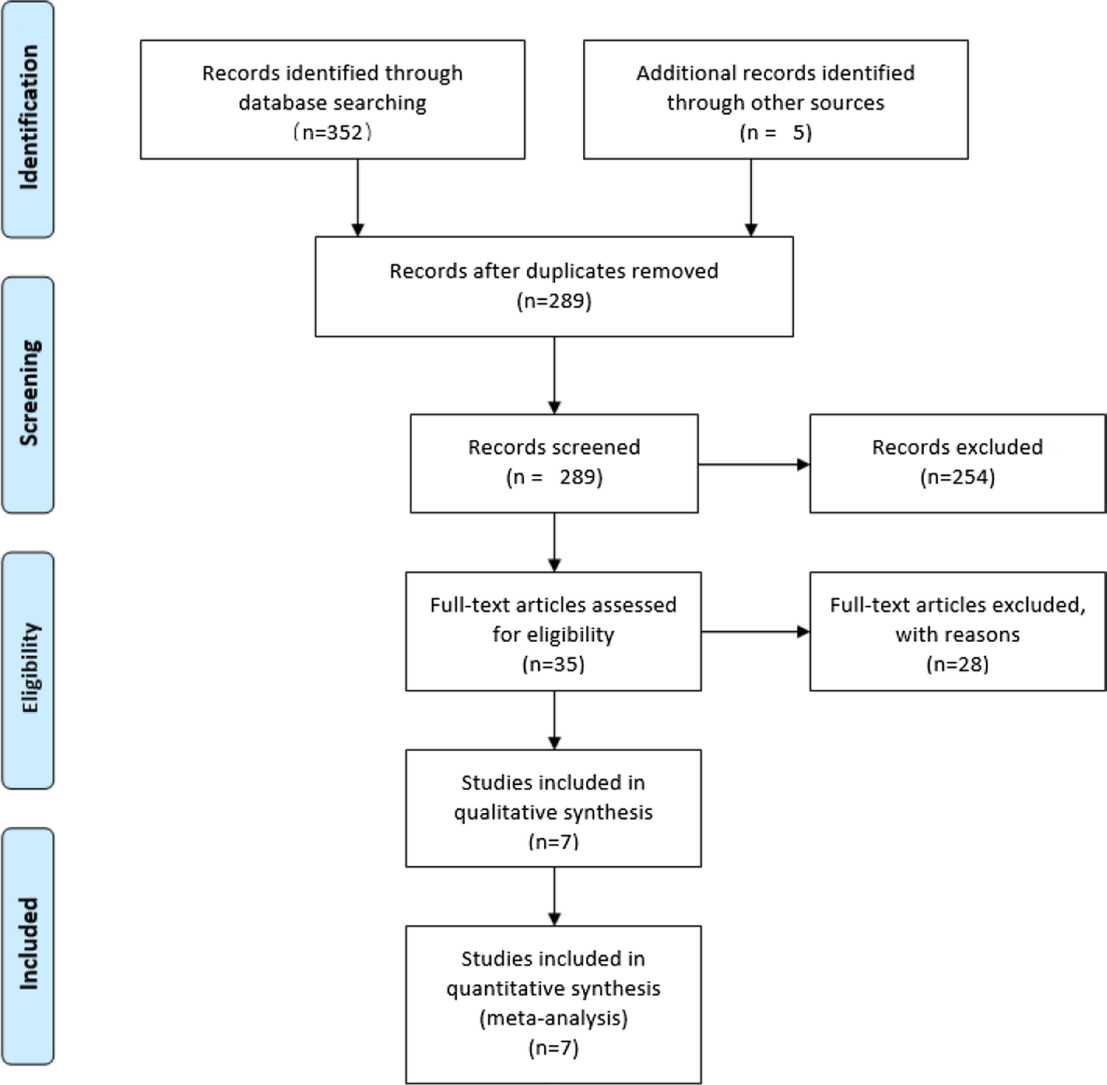
Study selection flowchart

As shown in Fig. 3 (study selection flowchart), 352 studies were searched from the database. Five articles have been added to the analysis through reference retrieval. After 68 repetitions were eliminated, 289 citations were screened for titles and abstracts, of which 254 citations were excluded from irrelevant subjects or research types. A total of 35 studies were to be reviewed. Among them, the excluded studies were used as meeting summaries, and 28 were excluded. Because of insufficient data or irrelevant results, the final 7 studies were included in the meta-analysis[21–27].

### Clinical characteristics of patients

All these seven studies were retrospective. The total number of patients was 820, of which 395 received HMAs before allo-HSCT, and the control group received BSC. The basic principle of treatment decision was not clearly stated in most articles. The decision was based on clinical conditions and doctors' judgment. However, according to the comparison of patients’ characteristics in seven studies, two of them showed that the median age of HMAs group was higher than that of the control group, and patients who chose the BSC for bridging treatment were more inclined to early transplantation.

### Analysis of outcomes

**Fig. 4:**
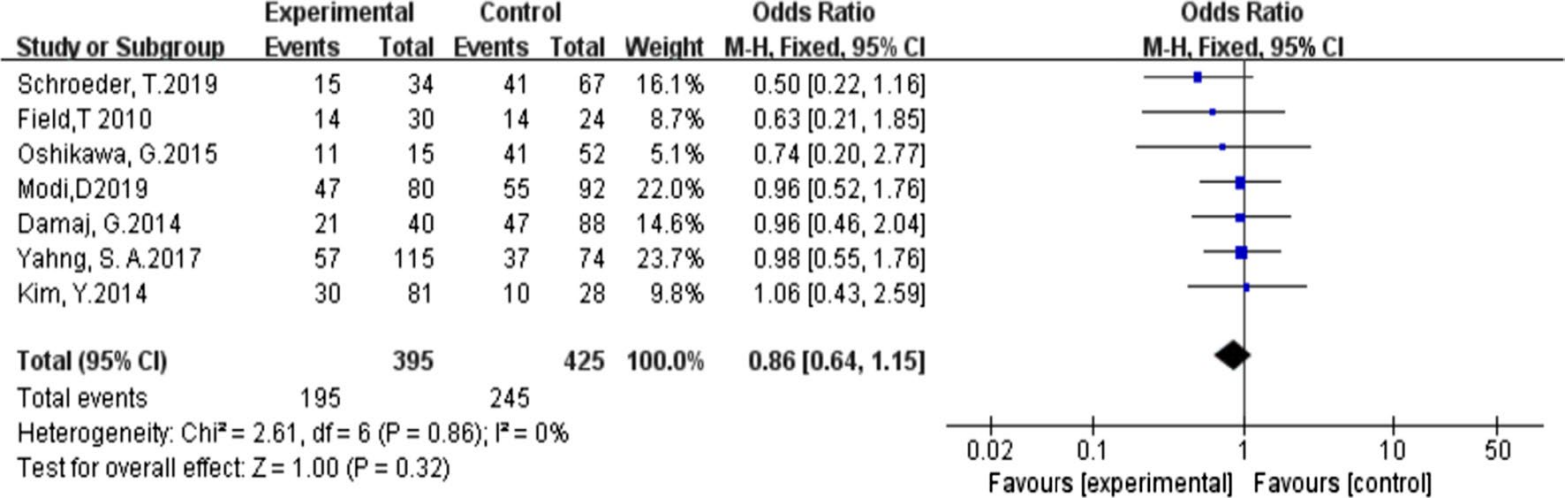
Forest plots

As shown in Fig. 4 (HR and 95% CI forest plots to assess OS with HMAs before allo-HSCT), all cohort studies compared the effectiveness of HMAs before allo-HSCT in MDS patients. Our results showed that administration of HMAs as pre-transplantation treatment did not improve OS (HR = 0.86, 95% CI: 0.64–1.15, *p* = 0.32), while the heterogeneity of the study was low (I^2 = 0%, *p* = 0.86).

## Discussion

In this study, we systematically analyzed all clinical studies in which HMAs were applied prior to allo-HSCT. It showed that MDS patients did not benefit from HMAs when subsequently underwent allo-HSCT. There was no significant improvement in OS after HS.

Although new drugs are constantly evolving, allo-HSCT remains the only treatment for cure which leads to complete and permanent eradication of the MDS aberrant clone, as evidenced by long-term hematopoiesis. Cytoreductive therapy prior to allo-HSCT is advised for patients who have ≥ 10% bone marrow myeloblasts [10]. While patients aged from 60 to 75 years old account for the majority of MDS patients. Primary endpoints for treatment of elderly MDS patients were maintaining a good quality of life rather than curative. Although MDS cannot be cured by HMAs, the emergence of hypomethylation treatment makes patients get better response, which is proved by stable blood count, transfusion independence and acceptable safety[6, 28–30]. In this sense, hypomethylation treatment is helpful for allo-HSCT, because it can obtain incalculable pre-transplant preparation time (donor seeking, financial support, etc.). TP53 mutations and complex karyotypes are more common in elderly patients. Some studies have shown that MDS/AML patients with TP53 mutations and/ or complex karyotypes have good initial response rates to decitabine [31–33]. It was also reported that AZA improved OS and relapse free survival (RFS) for higher-risk myelodysplastic syndrome (HR-MDS) patients with chromosome 7 abnormalities [34, 35]. This suggests that HMAs therapy before allo-HSCT may be effective in MDS patients with complex karyotype and/or TP53 mutations.

The adverse effect of HMAs cannot be ignored. Potential myelosuppression may increase the chance of infection and bleeding events, even life-threatening. All interventions aimed at reducing disease burden before allo-HSCT were likely to increase the risk of complications and inability to receive transplantation [36]. In these seven studies, many patients failed to accept allo-HSCT due to disease progression or treatment-related death.

Yahng et al*.* reported that, through pre-HSCT hypomethylating treatment (HMT), achieving bone marrow complete response (mCR) was significantly associated with superior 4-year disease-free survival (DFS) compared to no marrow response group (87.3% vs 10.7%, *p* < 0.001). This difference was also evident in OS (90.9% vs 8.6%, *p* < 0.001), cumulative incidences of relapse (CIR) (6.5% vs 45.4%, *p* < 0.001) and treatment-related mortality (TRM) (6.2% vs 43.9%, *p* < 0.001) [37]. Another study showed that the DFS of patients accepted HMAs as bridging treatment was slightly higher than that of the BSC group (71.2% vs 59.2%). It also compared AZA responders who reached CR, mCR, or SD with HI with non-responders, better OS (100% vs 66.7%, *p* = 0.066) and DFS (75% vs 50%, *p* = 0.305) were observed [26]. Voso et al. also found that AZA treatment before transplantation was beneficial if patients reached CR[38]. While no significant difference was found between pre-SCT AZA responders and non-responders in OS and RFS in another research [23]. There were also researches claimed that pre-transplantation therapy may favor the selection of resistant clones. Patients given the BSC had a higher likelihood to respond to HMAs as salvage therapy for relapse in comparison with patients undergoing bridging therapy[24]. The effect of HMAs on disease progression or relapse after transplantation was not significant in three articles. The CIR was comparable between the HMT group and the BSC group (6.2% vs 0%, 35% vs 36%, 31% vs 36%) [21–23]. Three studies reported the RFS of HMAs group was not better than that of BSC group (38% vs 38%, 37% vs 42%, 41% vs 51%) [22–24]. Similarly, Modi et al*.* concluded that no association was found between reduced post-transplant relapse and improved survival with the use of HMAs for cytoreductive therapy before allo-HSCT [27]. Interestingly, patients who received BSC performed as well as patients with blast counts < 5% and received HMAs. The patients who achieved cytoreduction with blasts counts < 5% fared better than ≥ 5% blast, which implies chemosensitivity and the biology of the disease affect the post-HSCT outcome, rather than the pre-transplantation treatment. Previous studies have also reported similar results[39, 40].

Besides, some pre-clinical studies have shown the anti-graft-versus-host disease (anti-GVHD) effects of AZA[41–43]. Among current retrospective clinical analysis, three studies showed that the use of pre-HSCT HMAs did not reduce the incidence of GVHD [21, 23, 26]. The timing of transplantation also plays a key role in the treatment and prognosis of MDS patients. For low and intermediate-1 risk MDS, delayed allo-HSCT is associated with maximum life expectancy, while for intermediate-2 and high-risk MDS, direct transplantation can obtain the maximum survival time [44]. This requires doctors to make decisions based on experience and family consultation.

Our research has a few limitations. First of all, different studies used different analytical models, which may explain the heterogeneity of seven studies. Second, the number of participants in eligible studies was too small to conduct subgroup analyses. In addition, there were many clinical heterogeneity in the study, such as the type of HMAs, the treatment of the control group, the age and gender, MDS subtypes, HLA differences, conditioning regime and follow-up time differences, any of these may affect the clinical results. In conclusion, our meta-analysis showed that HMAs as a bridging therapy before allo-HSCT did not improve the long-term survival. However, due to the limitations of the original studies, our conclusions need to be further verified by a larger sample and randomized controlled design prospective study. This will be instructive for the treatment of MDS patients.
